# Increased Prevalence of Metabolic Syndrome in Patients with Acne Inversa

**DOI:** 10.1371/journal.pone.0031810

**Published:** 2012-02-16

**Authors:** Robert Sabat, Akewit Chanwangpong, Sylke Schneider-Burrus, Deborah Metternich, Georgios Kokolakis, Agata Kurek, Sandra Philipp, Daniela Uribe, Kerstin Wolk, Wolfram Sterry

**Affiliations:** 1 Department of Dermatology and Allergy, University Hospital Charité, Berlin, Germany; 2 Interdisciplinary Group of Molecular Immunopathology, Dermatology/Medical Immunology, University Hospital Charité, Berlin, Germany; 3 Psoriasis Research and Treatment Center, Department of Dermatology, University Hospital Charité, Berlin, Germany; Aarhus University, Denmark

## Abstract

**Background:**

*Acne inversa* (AI; also designated as *Hidradenitis suppurativa*) is a common chronic inflammatory skin disease, localized in the axillary, inguinal and perianal skin areas that causes painful, fistulating sinuses with malodorous purulence and scars. Several chronic inflammatory diseases are associated with the metabolic syndrome and its consequences including arteriosclerosis, coronary heart disease, myocardial infraction, and stroke. So far, the association of AI with systemic metabolic alterations is largely unexplored.

**Methods and Findings:**

A hospital-based case-control study in 80 AI patients and 100 age- and sex-matched control participants was carried out. The prevalence of central obesity (odds ratio 5.88), hypertriglyceridemia (odds ratio 2.24), hypo-HDL-cholesterolemia (odds ratio 4.56), and hyperglycemia (odds ratio 4.09) in AI patients was significantly higher than in controls. Furthermore, the metabolic syndrome, previously defined as the presence of at least three of the five alterations listed above, was more common in those patients compared to controls (40.0% versus 13.0%; odds ratio 4.46, 95% confidence interval 2.02 to 9.96; *P*<0.001). AI patients with metabolic syndrome also had more pronounced metabolic alterations than controls with metabolic syndrome. Interestingly, there was no correlation between the severity or duration of the disease and the levels of respective parameters or the number of criteria defining the metabolic syndrome. Rather, the metabolic syndrome was observed in a disproportionately high percentage of young AI patients.

**Conclusions:**

This study shows for the first time that AI patients have a high prevalence of the metabolic syndrome and all of its criteria. It further suggests that the inflammation present in AI patients does not have a major impact on the development of metabolic alterations. Instead, evidence is given for a role of metabolic alterations in the development of AI. We recommend monitoring of AI patients in order to correct their modifiable cardiovascular risk factors.

## Introduction

Acne inversa (AI; also referred to as *Hidradenitis suppurativa*) is a chronic, destructive and scarring inflammatory skin disease with a prevalence of 1 to 4% [1], [2]. It mostly affects the intertriginous skin of perianal, inguinal, and axillary sites, however, submammary, periumbilical, retroauricular and nuchal sites can also be involved [3], [4]. Initially, infundibular hyperkeratosis and hyperplasia of the follicular epithelium leads to stasis in the hair follicle unit and formation of subcutaneous nodules [5]. Already at this early stage, a perifollicular infiltration of immune cells is present in AI lesions [6]. Immune cells by means of their mediators probably induce/enhance the infundibular hyperkeratosis and hyperplasia. Subsequently, the nodules rupture and/or meld, forming painful, deep dermal abscesses [3], [4]. The persistence of bacteria in obstructed and ruptured hair follicles supports the immune cell infiltration and inflammation and leads to a purulent exudate. In the late-stage, painful, fistulating sinuses and large indurated inflammatory plaques with extensive scarring emerge. Without treatment, the disease is chronic and progressive. AI has a great emotional impact on patients and causes social embarrassment as well as isolation [7], [8]. Currently, the treatment with the best curative prospect is the surgical intervention. Small lesions are usually be excised locally and primary closured [9], [10]. Larger lesions require the radical wide excision of the affected areas followed by reconstructive intervention [9], [11]. The use of antibiotics and tumor necrosis factor (TNF)-α blockers has been shown to improve symptoms without ensuring definite cure [12], [13], [14], [15]. Nonetheless, in 15–35% of patients TNF-α targeting caused long-lasting improvement (≥3 months) after the end of the therapy [12], [16], [17].

The etiology of AI remains enigmatic [3], [18]. Smoking, obesity, hormonal factors and a putative polygenic genetic background may play a role in its development and/or course. Very recently we demonstrated that AI lesions show a relative deficiency in the expression of anti-microbial proteins, which may contribute to the cutaneous bacterial persistence and following inflammation of the affected skin of AI patients [19]. Furthermore, we found in AI lesions a relative deficiency of interleukin (IL)-22 and IL-20 [19], two members of the IL-10 cytokine family [20], [21], [22]. IL-22 and IL-20 are known to up-regulate anti-microbial protein expression and are critical for the increase of these molecules under inflammatory conditions in human epidermis models [19], [23]. Interestingly, in AI lesions IL-22 deficiency was associated with increased IL-10 levels [19]. It seems quite likely that bacteria, the content of the ruptured hair follicle unit, and TNF-α induce IL-10 which inhibits the lymphocytic IL-22 production. Limited IL-22 production, in turn, leads to limited keratinocyte IL-20 production, and the reduced production of both cytokines seems to be responsible for deficient anti-microbial protein expression. This allows the bacterial persistence in AI lesions going along with strong, chronic inflammation and sustained IL-10 production.

Chronic inflammation in defined diseases, such as rheumatoid arthritis and psoriasis, is associated with metabolic and physiological alterations like central obesity, elevated blood pressure, increased levels of fasting blood glucose, elevated triglyceride (TG), and reduced high density lipoprotein (HDL)-cholesterol [24], [25], [26]. The coincidence of three or more of these abnormalities is called metabolic syndrome. The appearance of the metabolic syndrome is very important since it increases the risk of cardiovascular disorders such as arteriosclerosis, coronary heart disease, myocardial infarction, and stroke, leading to reduced life expectancy [27], [28], [29], [30]. However, there are no data regarding the prevalence of different criteria for metabolic syndrome in AI patients. Thereby, the aim of our current study was to investigate their frequency in patients suffering from AI.

## Methods

### Ethics Statement

This study involving human participants was accepted by the Charité ethics committee (*Ethikkommission der Charité Universitätsmedizin Berlin*). Written informed consent was obtained from all study participants.

### Study population

We performed a hospital-based case - control study with 80 patients suffering from AI and 100 control participants from the area around Berlin. All those AI patients were enrolled in the study, who (i) visited the Department of Dermatology, University Hospital Charité, Berlin, Germany, (ii) gave written informed consent, and (iii) fulfilled the following inclusion criteria: age of at least 18 years, absence of any malignant disorder, no previous treatment with immunosuppressive agents. As control participants were enrolled individuals who (i) replied to our announcement (e.g. via internet and posters) for the search of study participants, (ii) gave written informed consent, (iii) were older than 18 years and were matching in age and sex with the AI patients, and (iv) had never been diagnosed with AI and were not immunocompromised or diagnosed with any malignant disorder.

After obtaining informed consent, clinical data were collected (age, gender, weight, height, waist circumference, blood pressure, and history of illness). The body mass index (BMI) was calculated as weight (kg)/[height (m)]^2^. The waist circumference was measured at the level of the most upper part of the hipbone without compression on the skin. Blood pressure was measured after a 5 minute rest in sitting position. Venous fasting blood was taken for investigation of plasma glucose, triglycerides (TG), and HDL.

AI was clinically diagnosed according to the following criteria:

- typical lesions, i.e. deep-seated painful nodules: ‘blind boils’ in early lesions; abscess, draining sinus, bridged scars and ‘tombstone’ open comedones in secondary lesions,
- typical topography, i.e. in axillae, groin, perineal and perianal regions, buttocks,
- persistence and recurrence of lesions.

The severity of AI was measured by the ‘Sartorius Score’ [31] from the axillary and inguinal region as follows:

- anatomical region involved (axillary or inguinal region). 3 points per involved region,
- number and scores of lesions (abscesses, nodules, fistulas, scars or others). 2 points for each nodule, 4 points for each fistula, 1 point for each scar, 1 point for each “other”,
- the longest distance between 2 relevant lesions, i.e. nodules and fistulas, in each region, or size if only 1 lesion. 2 points if <5 cm; 4 points if 5 to 10 cm; 8 points if >10 cm,
- if lesions were clearly separated by normal skin in each region: 0 points; if not: 6 points.

The metabolic syndrome was diagnosed in the presence of three or more of the following criteria, according to the US National Cholesterol Education Program Adult Treatment Panel III (NCEP-ATP III) [32]:

- central obesity: waist circumference ≥102 cm (male), ≥88 cm (female),
- hypo-HDL-cholesterolemia: plasma HDL-cholesterol <40 mg/dl (male), <50 mg/dl (female),
- hypertriglyceridemia: plasma triglyceride ≥1.695 mmol/l (≥150 mg/dl),
- hypertension: blood pressure ≥130/85 mmHg or use of medication for hypertension,
- hyperglycemia: fasting plasma glucose ≥6.1 mmol/L (≥110 mg/dl) or use of medication for hyperglycemia.

### Statistical analysis

Standard descriptive statistics such as mean, standard deviation (SD), and standard error of the mean (SEM) were computed. Further statistical calculations were made using SPSS 14.0 software (SPSS). Results from AI patients and control participants were compared using the Mann–Whitney U test (two-tailed) or Chi-square test. *P*-values<0.05 were considered to be statistically significant. Correlation analysis was performed between severity of AI (Sartorius score), duration of AI, number of positive metabolic syndrome criteria, and separate diagnostic parameters of metabolic syndrome by means of Spearman's rank correlation analysis.

## Results

In our current study we investigated the levels of metabolic syndrome parameters and the prevalence of fulfilled respective criteria in 80 patients suffering from AI and compared these to results from 100 respective control participants. As a prerequisite for these analyses, there were no differences in age and sex between these two groups. In fact, the sex ratio was approximately 1∶1, and their average age was ∼40 years in both cohorts (**Table 1**).

**Table 1:** Demographic characteristics of AI patients and controls.

	AI patients	Controls	*P*-value
Males (%)	46.3%	44.0%	0.763
Females (%)	53.7%	56.0%	0.763
Age in years			
(mean ± SD)	40.0±10.6	40.9±10.5	0.639
(range)	21–62	20–63	

The *P*-values calculated by the Chi-square test (sex distribution) or Mann–Whitney U test (age) are indicated.

In the patient group, the disease duration and the Sartorius scores had a rather wide range, namely from 1 to 41 years and from 12 to 84, respectively (**Table S1**), which facilitated the analysis of the possible interrelation between these parameters and the magnitude of various metabolic alterations. The mean disease duration was 11 years in male and 13 years in female AI patients. The average Sartorius score was about 35 in both male and female patients. Roughly one half of the patients never had any surgical intervention at the time of enrolment.

**Figure 1:**
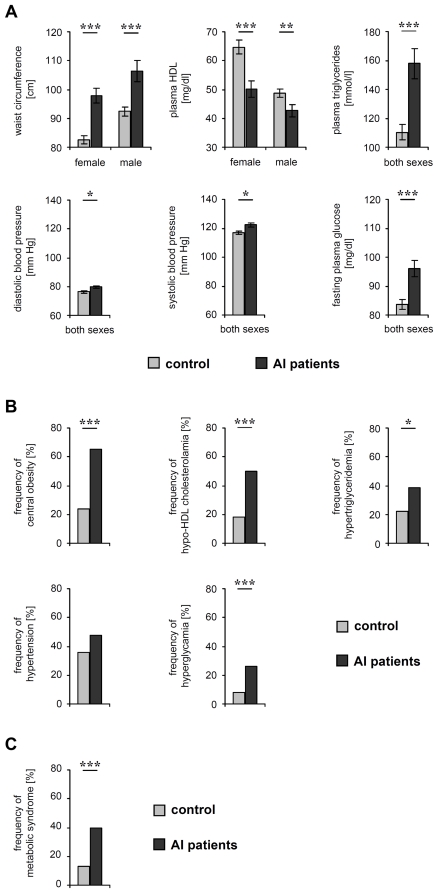
Parameter levels and frequency of fulfilled criteria for metabolic syndrome in AI patients and control persons. (A) The average waist circumference, plasma HDL-cholesterol and TG levels, systolic and diastolic blood pressure, and fasting plasma glucose levels in AI patients and control participants are demonstrated as the mean ± SEM. Significance of differences was assessed by the Mann–Whitney U-test (*P<0.05, **P<0.01, ***P<0.001). (B) The frequency of central obesity, hypo-HDL-cholesterolemia, hypertriglyceridemia, hypertension, hyperglycemia in AI patients and controls are given. Significance of differences was assessed by the Chi-square test (*P<0.05, **P<0.01, ***P<0.001). (C) The percentages of AI patients and controls with metabolic syndrome are given. Significance of differences was assessed by the Chi-square test (**P<0.01).

**Figure 2:**
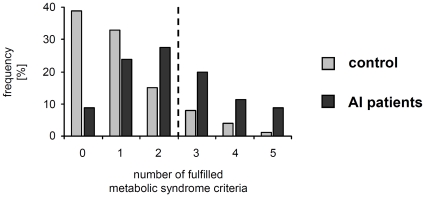
Percentages of AI patients and control participants that fulfilled the indicated number of metabolic syndrome criteria. The dashed vertical line marks the cutoff for the metabolic syndrome.

The quantification of metabolic syndrome parameters in AI patients and controls uncovered that average waist circumference, plasma TG levels, fasting plasma glucose levels, as well as the systolic and diastolic blood pressure were significantly higher in AI patients than in control participants (**Figure 1** **, Table S2**). Furthermore, the average plasma HDL levels were lower in AI patients. Even more importantly, the prevalence of central obesity (odds ratio 5.88; 95% confidence interval, 2.93 to 11.91, *P*<0.001), hypertriglyceridemia (odds ratio 2.24; 95% confidence interval, 1.11 to 4.54, *P*<0.014), hypo-HDL-cholesterolemia (odds ratio 4.56; 95% confidence interval, 2.21 to 9.46, *P*<0.001), and hyperglycemia (odds ratio 4.09; 95% confidence interval, 1.59 to 10.84, *P*<0.001) in AI patients were significantly higher than in control participants (**Figure 1** **, Table S2**). Finally, the metabolic syndrome was significantly more common in the patients than in controls (40.0% *versus* 13.0%, *P*<0.001, odds ratio 4.46; 95% confidence interval, 2.02 to 9.96) (**Figure 1** **, Table S2**). Additionally, 20% of all AI patients (half of the AI patients with metabolic syndrome) compared to only 5% of all controls (38.5% of controls with metabolic syndrome) were positive for more than three of the metabolic syndrome-defining criteria (**Figure 2**) and, therefore, had an even higher risk for cardiovascular diseases [27].

**Figure 3:**
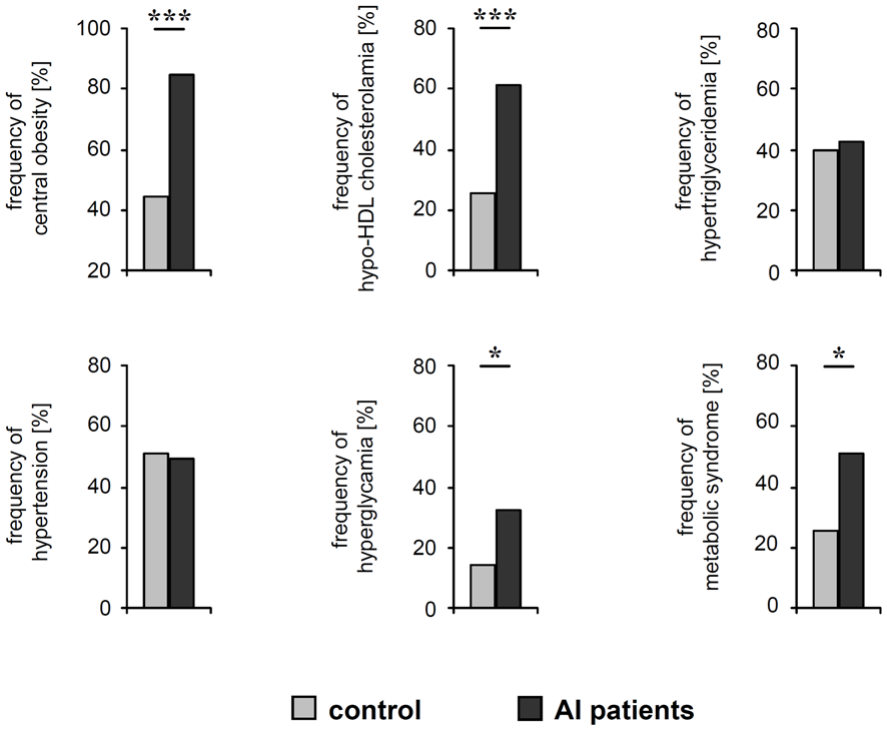
Frequency of fulfilled criteria for metabolic syndrome in overweight and obese AI patients and control participants. The frequency of central obesity, hypo-HDL-cholesterolemia, hypertriglyceridemia, hypertension, hyperglycemia in AI patients (n = 59) and controls (n = 43) with a BMI of 25 or higher are given. Significance of differences was assessed by the Chi-square test (*P<0.05, ***P<0.001).

The analysis of subgroups such as overweight individuals (BMI≥25) demonstrated that the prevalence of metabolic syndrome and its parameters were significantly higher in respective patients than in respective controls (for metabolic syndrome 50.8% *versus* 25.6%, *P*<0.01, odds ratio 3.01; 95% confidence interval, 1.18 to 7.76) (**Figure 3**). We also observed a two times higher prevalence of metabolic syndrome in smoking AI patients compared to smoking control participants (data not shown). However, because the smoking controls are a small population whereas about 90% of AI patient are smokers, further investigations are necessary to support this conclusion.

**Figure 4:**
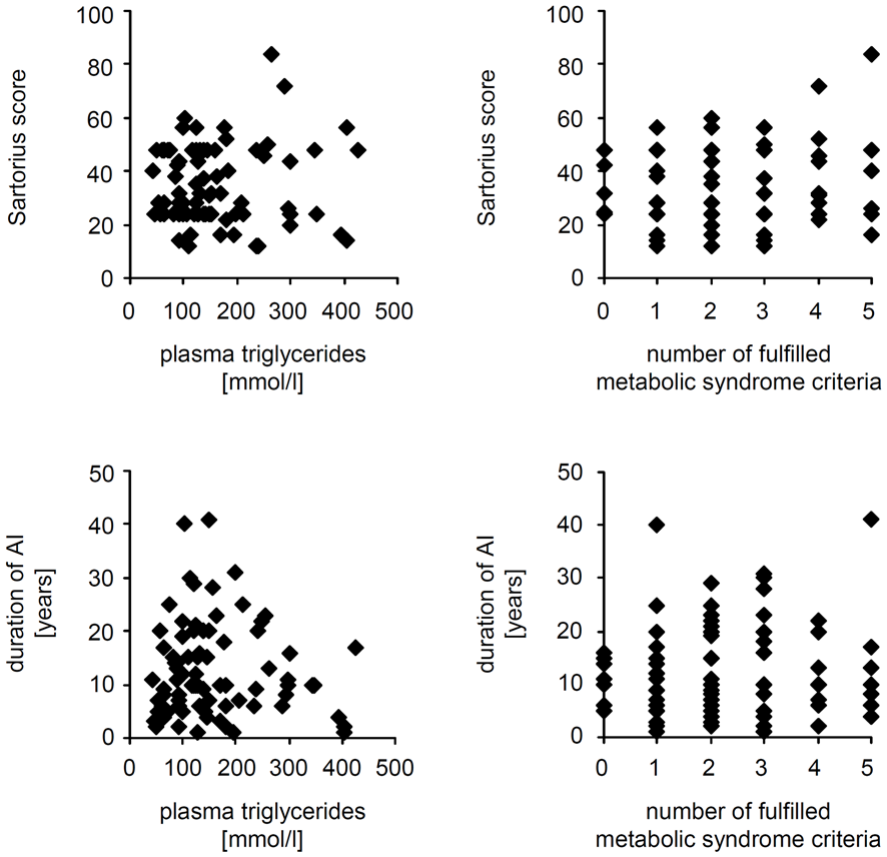
Correlation of disease severity and duration with parameters of the metabolic syndrome for AI patients. The correlation of Sartorius scores and duration of AI with each plasma TG levels and the number of positive metabolic syndrome criteria was investigated by Spearman's rank correlation analysis. No significant correlation was found.

These results raised the question about the mechanisms underlying the increased frequency of the metabolic syndrome in AI patients. The simplest hypothesis may be that the chronic inflammation present in AI patients promotes and enhances metabolic alterations. If this hypothesis was correct then the severity and/or duration of disease would positively correlate with the magnitude of the metabolic alterations. To test this, we analyzed the correlation between the severity and duration of the illness and the waist circumference, plasma TG and HDL levels, diastolic and systolic blood pressure, fasting plasma glucose levels, and the number of fulfilled criteria for metabolic syndrome. Surprisingly, no significant correlations between the analyzed parameters were found (**Figure 4** **,** **Table 2**). Furthermore, we did not find any significant correlations between the levels of analyzed parameters and the age at AI diagnosis (**Table 2**). Next, we subdivided the AI patients in two subgroups, namely those with relatively mild disease (Sartorius score <30; n = 39) and those with severe symptoms (Sartorius score >30; n = 41). Again, there was no difference in the levels of metabolic parameters and no difference in the prevalence of central obesity, hypertriglyceridemia, hypo-HDL-cholesterolemia, arterial hypertension, hyperglycemia, or metabolic syndrome between these two groups (data not shown).

**Table 2:** Correlation of severity and duration of AI with parameters of metabolic syndrome.

	Sartorius score	Duration	Age at AI diagnosis
Waist circumference (cm)	0.060(0.600)	−0.082(0.470)	0.011(0.924)
Plasma HDL-cholesterol (mg/dl)	−0.072(0.527)	0.118(0.299)	0.142(0.208)
Plasma TG (mmol/l)	−0.048(0.673)	0.058(0.609)	0.124(0.275)
Diastolic blood pressure (mmHg)	0.196(0.082)	0.052(0.644)	0.007(0.949)
Systolic blood pressure (mm Hg)	0.143(0.205)	−0.034(0.764)	0.056(0.621)
Fasting plasma glucose (mmol/l)	0.079(0.486)	−0.032(0.776)	−0.063(0.582)
BMI (kg/m^2^)	0.172(0.128)	−0.100(0.379)	−0.118(0.299)
Number of fulfilled metabolic syndrome criteria	0.042(0.711)	0.050(0.663)	−0.101(0.663)

The correlation was investigated by Spearman's rank correlation analysis. For each field, the Spearman's rank correlation coefficient and, in parenthesis, the *P*-values are indicated.

Because of this unexpected finding we started further analysis and explored the possibility whether surgical treatment with reduction of the inflammatory load might reduce manifestations of the metabolic syndrome. We compared the prevalence of different criteria for metabolic syndrome and the levels of respective parameters in 38 patients that never had surgical intervention (pre-operative patients) and versus 42 patients that already had at least one surgical intervention (post-operative patients). There were no significant differences in sex, age, and duration of disease between these two groups (**Table S3**). As expected, the Sartorius score was lower in the post-operative group as compared to the pre-operative group. However, no differences for any of the criteria for the metabolic syndrome between both groups could be detected (**Table 3**). To substantiate this finding, we analyzed details about further 12 patients (5 males, 7 females) who had their last surgical intervention averagely 4 years ago (199±15 weeks). Although these patients had no or minimal disease activity, their current BMI had remained unchanged compared to values before surgery (26.8 *versus* 26.3). All these data suggest that the inflammation present in AI patients does not have any major impact on their metabolic alterations.

**Table 3:** Criteria and parameters of metabolic syndrome in pre-operative and post-operative patients suffering from AI.

	Pre-operativeAI patients	Post-operativeAI patients	*P*-value
Waist circumference (cm)			
female	100.4±16.3	94.8±16.7	0.542
male	98.1±25.0	112.0±18.7	**0.009**
Central obesity (%)	57.9%	71.4%	0.207
Plasma HDL-cholesterol (mg/dl)			
female	49.5±17.8	51.0±19.5	0.836
male	39.0±7.1	45.2±15.3	0.505
Hypo-HDL-cholesterolemia (%)	55.3%	45.2%	0.370
Plasma TG (mmol/l)	152.3±95.7	162.7±92.6	0.424
Hypertriglyceridemia (%)	36.8%	40.5%	0.739
Blood pressure (mm Hg)			
diastolic	79.5±6.3	80.0±9.0	0.860
systolic	121.7±7.7	123.0±14.2	0.744
Hypertension (%)	44.7%	50.0%	0.638
Fasting plasma glucose (mmol/l)	101.4±31.2	91.3±18.2	0.161
Hyperglycemia (%)	23.7%	28.6%	0.620
BMI (kg/m^2^)	29.1±7.6	30.2±7.3	0.458
Metabolic syndrome (%)	34.2%	45.2%	0.315
Sartorius score	39.0±15.2	31.0±13.4	**0.014**

The percentages of pre-operative and post-operative patients with pathological alterations (%) and mean ± SD data of the analyzed parameters are shown. The *P*-values calculated by the Mann–Whitney U-test (for waist circumference, plasma HDL-cholesterol, plasma TG, blood pressure, fasting plasma glucose) or thy Chi-square test (for frequency of central obesity, hypo-HDL-cholesterolemia, hypertriglyceridemia, hypertension, hyperglycemia, and metabolic syndrome) are indicated. Significant *P*-values (<0.050) are in boldface.

**Figure 5:**
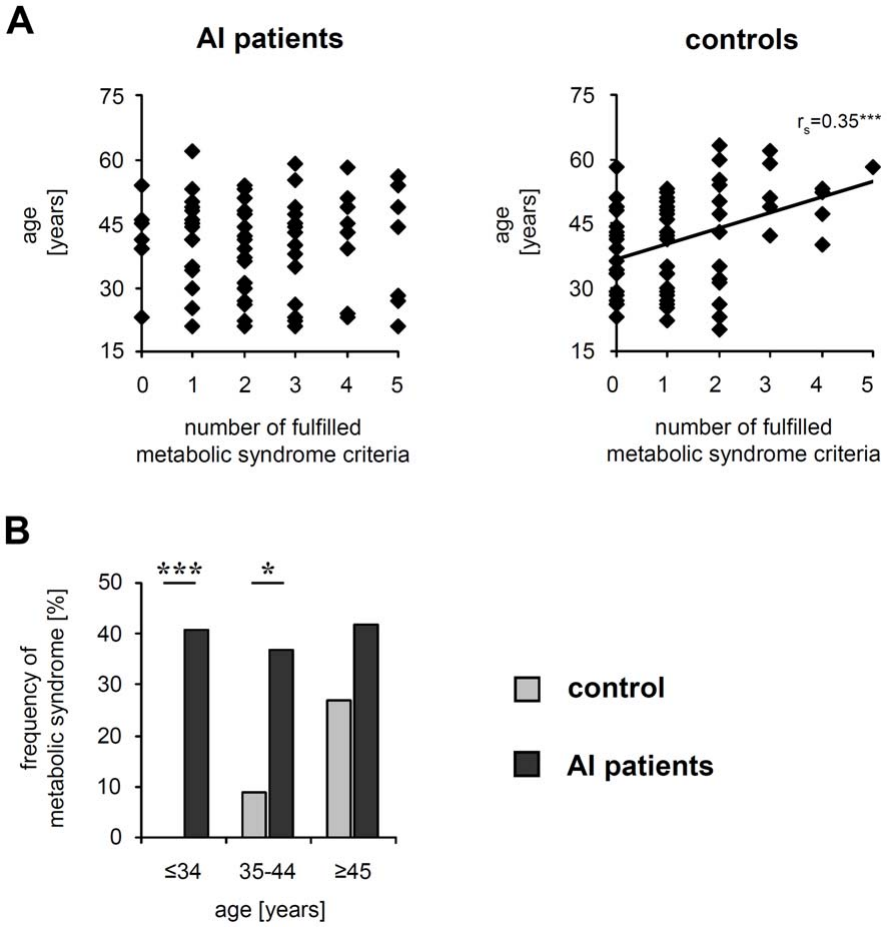
Correlation between age and metabolic syndrome for AI patients and control participants. (A) The correlation of age with the number of fulfilled metabolic syndrome criteria was investigated by Spearman's rank correlation analysis for AI patients and controls. Significant correlation was found only for controls (Spearman's rank correlation coefficient r_s_ = 0.363, ***P<0.000). (B) The prevalence of the metabolic syndrome in AI patients and control participants in different age groups is given (≤34 years old AI patients: n = 22; ≤34 years old control participants: n = 36; 35 to 44 years old AI patients: n = 27, 35 to 44 years old control participants: n = 23; ≥45 years old AI patients: n = 31; ≥45 years old control participants: n = 41). Significance of differences was assessed by the Chi-square test (*P<0.05, ***P<0.001).

In the last part of our study we questioned which AI patients are particularly endangered by the metabolic syndrome. The frequency of the metabolic syndrome usually increases with increasing age of a population. Surprisingly, we found no significant correlation between the patients' age and the number of fulfilled metabolic syndrome criteria or between the age and respective parameter levels even if such correlations clearly existed in our control population (**Figure 5A** **,** **Table 4**). In order to understand the cause of the non-existing correlations with the age in AI patients, we subdivided these patients and controls into three age groups: ≤34-year-olds, 35 to 44-year-olds, and ≥45-year-olds, and determined the prevalence of metabolic syndrome in each group. By doing so we surprisingly found no differences between these three age groups in AI patients. Rather, in contrast to the control participants, already very young AI patients were detected to suffer from the metabolic syndrome (**Figure 5B**). In fact, the odds ratios for the prevalence of metabolic syndrome calculated for each age group of AI patients were: >20 (≤34-year-olds), 6.18 (35 to 44-year-olds), and 1.97 (≥45-year-olds), even if they were based on small groups (between 22 and 41 persons per group). These data provided strong evidence that the appearance of the metabolic syndrome affects a disproportionately high number of young AI patients.

**Table 4:** Correlation of different parameters of metabolic syndrome with the age of AI patients and controls.

	AI patients	Controls
Waist circumference (cm)	0.050(0.661)	0.433(**0.000**)
Plasma HDL-cholesterol (mg/dl)	0.235(**0.036**)	−0.126(0.213)
Plasma TG (mmol/l)	0.171(0.129)	0.166(0.099)
Diastolic blood pressure (mm Hg)	0.094(0.406)	0.261(**0.009**)
Systolic blood pressure (mmHg)	0.113(0.319)	0.261(**0.009**)
Fasting plasma glucose (mmol/l)	−0.051(0.652)	0.331(**0.001**)
BMI (kg/m^2^)	−0.111(0.327)	0.353(**0.000**)
Number of fulfilled metabolic syndrome criteria	0.012(0.916)	0.363(**0.000**)

The correlation was investigated by Spearman's rank correlation analysis. For each field, the Spearman's rank correlation coefficient and, in parenthesis, the *P*-values are indicated. Significant *P*-values (<0.050) are in boldface.

## Discussion

In our current study we investigated the prevalence of the metabolic syndrome in patients suffering from AI and demonstrated for the first time a significantly higher frequency of metabolic syndrome in such patients compared to age- and sex-matched controls. Accordingly, the prevalence of central obesity, hypo-HDL-cholesterolemia, hypertriglyceridemia, and hyperglycemia was also significantly elevated in AI patients. Finally, the average waist circumference, plasma TG levels, fasting plasma glucose levels, as well as the systolic and diastolic blood pressure were significant higher, and average plasma HDL levels were lower in AI patients than in control participants.

Commonly, these metabolic and physiological alterations increase the risk of coronary heart disease, myocardial infarction, and stroke leading to reduced life expectancy [27], [28], [29], [30]. That implies that 40% of our AI patients are considerably endangered by these cardiovascular diseases. This is of particular importance since the endangering also affects very young AI patients. In fact, approximately 40% of our AI patients that were younger than 35 years suffered from metabolic syndrome compared to 0% of respective age- and sex-matched controls (odds ratio >20). In the age group between 35 and 44 years the difference, although being slightly lower, was still very high (odds ratio 6.18). We believe that an important message of our study is that clinicians who treat AI patients take into consideration that (i) a large portion of their patients have metabolic problems that are indicated neither by the Sartorius score nor by the duration of the disease, but that need to be addressed and (ii) that even very young AI patients are affected with this.

Increased prevalence of the metabolic syndrome is also known from patients suffering from some other chronic inflammatory diseases, e.g., psoriasis [33]. However, there seem to be certain differences between psoriasis and AI in this regard. First, the prevalence of metabolic disturbances [34], [35] and metabolic syndrome [26], [36] in AI patients appears to be higher than in psoriasis patients. For example, Love et al. very recently showed for populations of psoriasis patients and controls, which, regarding age and sex, were comparable to our cohort, a prevalence of metabolic syndrome of 31.4% and 17.1%, respectively, and an odds ratio of 2.22 [36]. Second, in psoriasis [26] but not in AI patients there was an association between disease duration and metabolic syndrome appearance and between at least some criteria of the metabolic syndrome and the severity of the disease. Third, the metabolic syndrome preferentially affects psoriasis patients at a mostly higher age [26], whereas many young AI patients are concerned. Thereby, the consequences of metabolic alterations for AI patients could be even markedly worse than for psoriasis patients, for whom increased mortality from cardiovascular diseases has been documented [37].

After finding that AI patients suffer more frequently from the metabolic syndrome we asked for the underlying mechanism. To test the possibility that the chronic inflammation induces metabolic disturbances in these patients, we correlated Sartorius score and duration of disease with individual parameters for metabolic syndrome. Surprisingly, no significant correlation was found. Interestingly, with the exception of the Sartorius score we also did not detect significant differences regarding metabolic alterations between AI patients who never had surgical intervention *versus* those after relevant surgical therapy. All these facts suggest that chronic inflammation is not the major driver of the metabolic alterations in AI patients. Based on our results we speculate that the metabolic alterations might be the primary rather than a secondary pathological event in these patients, i.e., that they might trigger AI. In fact, the metabolic alterations can lead to poor blood circulation of respective skin areas. Hypoxia in turn induces IL-10 production in CD4+ T-cells [38], and IL-10 inhibits the production of IL-22 [19], an important inducer of keratinocyte IL-20 production [19], [39]. Since IL-22 and IL-20 are major inducers of antibacterial proteins in epithelia [23], [40], [41], poor blood circulation would eventually lead to enhanced cutaneous bacterial persistence and the outbreak of AI. This hypothesis should be investigated by future studies.

## Supporting Information

Table S1 **Disease-related characteristics of AI patients.**
(DOC)

Table S2 **Parameters and criteria of metabolic syndrome in patients suffering from AI and in control participants.** The percentages of AI patients and control participants with pathological alterations (%) and mean ± SD data of the analyzed parameters are shown. The *P*-values calculated by the Mann–Whitney U-test (for waist circumference, plasma HDL-cholesterol, plasma TG, blood pressure, fasting plasma glucose) or the Chi-square test (for the frequency of central obesity, hypo-HDL-cholesterolemia, hypertriglyceridemia, hypertension, hyperglycemia, and metabolic syndrome) are indicated. Significant P-values (<0.050) are in boldface.
(DOC)

Table S3 **Demographic and disease-related characteristics of pre-operative and post-operative patients suffering from AI.** The *P*-values calculated by the Chi-square test (sex distribution) or the Mann–Whitney U-test (age and duration) are indicated.
(DOC)
