# Supplementary material for: Increased Prevalence of Metabolic Syndrome in Patients with Acne Inversa
Source: PLoS One. 2012 Feb 16;7(2):e31810. doi: 10.1371/journal.pone.0031810 (PMC3281019; doi:10.1371/journal.pone.0031810)
Supplement: Table S1 — Disease-related characteristics of AI patients. (DOC) [file pone.0031810.s001.doc]

Table S1

|  | Female  AI patients | Male  AI patients | Total |
| --- | --- | --- | --- |
| Duration of AI in years  (mean ± SD)  (range) | 13.7 ± 9.0  1 - 41 | 11.4 ± 8.5  1 - 31 | 12.7 ± 8.8  1 - 41 |
| Sartorius score  (mean ± SD)  (range) | 35.3 ± 14.4  12 - 84 | 34.3 ± 15.2  12 - 72 | 34.8 ± 14.7  12 - 84 |
| Surgical status  pre-operative (%)  post-operative (%) | 55.8%  44.2% | 37.8%  62.2% | 47.5%  52.5% |
| BMI  (mean ± SD)  (range) | 29.7 ± 7.4  19.3 - 49.7 | 29.7 ± 7.7  19.3 - 52.6 | 29.7 ± 7.45  19.3 - 52.6 |
| Weight status  underweight (%)  normal (%)  overweight (%)  obese (%) | 0%  27.9%  27.9%  44.2% | 0%  27.0%  35.1%  37.8% | 0%  27.5%  31.3%  41.3% |
