# Supplementary material for: Increased Prevalence of Metabolic Syndrome in Patients with Acne Inversa
Source: PLoS One. 2012 Feb 16;7(2):e31810. doi: 10.1371/journal.pone.0031810 (PMC3281019; doi:10.1371/journal.pone.0031810)
Supplement: Table S2 — Parameters and criteria of metabolic syndrome in patients suffering from AI and in control participants. The percentages of AI patients and control participants with pathological alterations (%) and mean ± SD data of the analyzed parameters are shown. The P-values calculated by the Mann–Whitney U-test (for waist circumference, plasma HDL-cholesterol, plasma TG, blood pressure, fasting plasma glucose) or the Chi-square test (for the frequency of central obesity, hypo-HDL-cholesterolemia, hypertriglyceridemia, hypertension, hyperglycemia, and metabolic syndrome) are indicated. Significant P-values (<0.050) are in boldface. (DOC) [file pone.0031810.s002.doc]

**Table S2**

|  | AI patients | Controls | *P*-values |
| --- | --- | --- | --- |
| Waist circumference (cm)  female  male | 97.8 ± 16.5  106.4 ± 22.2 | 82.5 ± 10.9  92.5 ± 10.2 | **0.000**  **0.000** |
| Central obesity (%) | 65.0% | 24.0% | **<0.001** |
| Plasma HDL (mg/dl)  female  male | 50.2 ± 18.4  42.7 ± 12.9 | 64.6 ± 18.0  48.8 ± 9.6 | **0.000**  **0.002** |
| Hypo-HDL-cholesterolemia (%) | 50.0% | 18.0% | **<0.001** |
| Plasma TG (mmol/l) | 157.8 ± 93.6 | 110.4% ± 55.6 | **0.000** |
| Hypertriglyceridemia (%) | 38.8% | 22.0% | **0.014** |
| Blood pressure (mm Hg)  diastolic blood pressure  systolic blood pressure | 79.8 ± 7.8  122.4 ± 11.5 | 76.3 ± 9.7  117.0 ± 13.4 | **0.029**  **0.016** |
| Hypertension (%) | 47.5% | 36.0% | 0.119 |
| Fasting plasma glucose (mmol/l) | 96.1 ± 25.6 | 83.7 ± 16.4 | **0.000** |
| Hyperglycemia (%) | 26.3% | 8.0% | **<0.001** |
| BMI (kg/m^2^) | 29.7 ± 7.5 | 24.3 ± 3.5 | **0.000** |
| Metabolic syndrome (%) | 40.0% | 13.0% | **<0.001** |
