# Supplementary material for: Increased Prevalence of Metabolic Syndrome in Patients with Acne Inversa
Source: PLoS One. 2012 Feb 16;7(2):e31810. doi: 10.1371/journal.pone.0031810 (PMC3281019; doi:10.1371/journal.pone.0031810)
Supplement: Table S3 — Demographic and disease-related characteristics of pre-operative and post-operative patients suffering from AI. The P-values calculated by the Chi-square test (sex distribution) or the Mann–Whitney U-test (age and duration) are indicated. (DOC) [file pone.0031810.s003.doc]

Table S3

|  | Pre-operative  AI patients | Post-operative  AI patients | *P*-value |
| --- | --- | --- | --- |
| Males (%) | 39.5% | 52.4% | 0.248 |
| Females (%) | 60.5% | 47.6% | 0.248 |
| Age in years  (mean ± SD)  (range) | 39.2 ± 10.0  21 - 62 | 40.8 ± 11.2  21 - 59 | 0.396 |
| Duration of AI in years  (mean ± SD)  (range) | 13.3 ± 9.8  1 - 41 | 12.1 ± 7.8  1 - 31 | 0.717 |
